# Longitudinal monitoring of adalimumab levels and anti-adalimumab antibodies in Chinese patients with ankylosing spondylitis: a prospective cohort study

**DOI:** 10.3389/fimmu.2026.1893483

**Published:** 2026-10-01

**Authors:** Xiaoliang Ding, Ling Xue, Mingjun Wang, Shizheng Pan, Bikui Zhang, Fen Li, Yingchun Ma, Nan Hu, Dongyi He, Lin Song, Sheng Jiang, Shengxiong Zhu, Jian Wu, Liyan Miao

**Affiliations:** 1 Department of Pharmacy, The First Affiliated Hospital of Soochow University, Suzhou, China; 2 Department of Rheumatology, The First Affiliated Hospital of Soochow University, Suzhou, China; 3 Department of Pharmacy, The Second Xiangya Hospital, Central South University, Changsha, China; 4 Department of Rheumatology and Immunology, The Second Xiangya Hospital, Central South University, Changsha, China; 5 Department of Rheumatology and Immunology, The Third Affiliated Hospital of Soochow University, Changzhou, China; 6 Department of Pharmacy, The Third Affiliated Hospital of Soochow University, Changzhou, China; 7 Department of Rheumatology, Shanghai Guanghua Hospital, Shanghai University of Traditional Chinese Medicine, Shanghai, China

**Keywords:** adalimumab, ankylosing spondylitis, anti-drug antibodies, drug monitoring, exposure-response

## Abstract

**Objective:**

This study aimed to clarify the association between longitudinal adalimumab concentrations, anti-adalimumab antibodies (AAA) levels, and clinical response in Chinese patients with ankylosing spondylitis (AS).

**Methods:**

A prospective multi-center, observational cohort study of AS patients receiving adalimumab therapy was conducted. Disease activity was assessed with the AS Disease Activity Score (ASDAS), and clinical responders were defined based on ASDAS improvement together with C-reactive protein normalization. Adalimumab concentrations were measured using a validated sandwich immunoassay. Measurement of AAA levels was performed with a laboratory-developed immunoassay system integrating electrochemiluminescence technology with specific immunomagnetic separation, with result reported as signal-to-noise ratio (S/N).

**Results:**

A total of 140 patients receiving adalimumab monotherapy were included. The median follow-up period was 169 days, and 711 plasma samples were available for adalimumab concentrations and AAA levels measurements. Seven patients had pre-existing AAA, and 120 (90.2%) of the remaining 133 patients tested positive for AAA during follow-up. A total of 48 patients were excluded from the exposure–response relationship dataset owing to pre-existing AAA (n=7), lack of active disease (n=26), incomplete follow-up (n=8), and prolonged dosing intervals (n=7). Among the remaining 92 patients, a total of 58 (63%) achieved clinical response. Adalimumab concentrations were higher, and AAA levels were significantly lower, in responders compared with non-responders at multiple time points. Exposure–response relationship models also revealed a steady-state adalimumab concentration target of 5.1 μg/mL and a week-4 concentration target of 3.3 μg/mL, corresponding to an 80% probability of clinical response. Thirty-six of 92 patients (39.1%) exhibited AAA-S/N values exceeding the putative clinically meaningful threshold (AAA-S/N=10). Patients in the high-AAA group (AAA-S/N≥10) had lower drug levels (median: 1.5 μg/mL vs. 6.9 μg/mL, *P<*0.0001) and poorer response rates (38.8% vs. 78.6%, *P* = 0.0002), relative to those in the low-AAA group (AAA-S/N<10). Furthermore, patients who were week-4 AAA-positive exhibited a 1.8-fold increased risk of non-response (relative risk, 95% confidence interval, 1.1–2.8).

**Conclusions:**

Our results demonstrate that longitudinal profiles of adalimumab concentrations and AAA levels show a strong association with clinical responses in this Chinese AS cohort, which may inform therapeutic drug monitoring–guided adalimumab treatment strategies that remain to be further validated.

**Trial registration:**

ClinicalTrials.gov (NCT04875299).

## 1 Background

Adalimumab, a tumor necrosis factor (TNF) inhibitor, plays a crucial role in the treatment of numerous autoimmune diseases, including rheumatoid arthritis, psoriasis, ankylosing spondylitis (AS), and Crohn’s disease (1). Based on existing data, adalimumab concentrations and clinical responses vary considerably between patients, posing a challenge (2, 3). Therapeutic drug monitoring (TDM) may help manage patients receiving adalimumab. Despite its potential benefits, the recommendations for implementing TDM for adalimumab treatment vary considerably across clinical guidelines and consensus statements (4–6).

The existing evidence supporting TDM of adalimumab comes from the gastroenterology field; evidence in inflammatory rheumatic and musculoskeletal diseases remains rather scarce. Thus, providing recommendations for these diseases is challenging (6). Notably, different diseases may have distinct requirements for therapeutic concentrations due to the heterogeneity in disease pathogenic mechanisms. In AS, an obvious exposure–response relationship and the optimal drug concentration range or target remain unknown. Meanwhile, adalimumab TDM can be categorized as either reactive or proactive (7). Reactive TDM is performed in cases of inadequate clinical response or disease flare to identify the underlying contributors. Proactive TDM is defined as a pre-scheduled monitoring scheme based on periodic measurements and regimen adjustments, irrespective of disease activity. Reactive TDM may be considered and is recommended in some settings; however, the clinical value of proactive TDM remains poorly defined. Longitudinal clinical data therefore offer a valuable resource for formulating feasible proactive TDM strategies.

Immunogenicity, defined as the unwanted immune response against therapeutic proteins and predominantly presenting with anti-drug antibodies (ADA) formation, limits the widespread use of these therapies (8). Adalimumab, a representative therapeutic monoclonal antibody used to treat various inflammation-mediated diseases, carries notable clinical concerns related to its inherent immunogenicity. Repeated adalimumab administration elicits anti-adalimumab antibodies (AAA) production in some patients, leading to accelerated drug clearance and treatment failure (9). Unfortunately, AAA measurements and interpretation of the AAA results in clinical practice pose challenges (3, 10). AAA measurement and reporting have not been standardized due to bioanalytical technical issues, including drug interference, poor assay sensitivity, and the lack of specific reference standards (11). Furthermore, clinically meaningful AAA levels are lacking (12). We previously developed a highly sensitive, drug-tolerant immunoassay platform and comprehensively characterized the ADA against adalimumab (13). A promising AAA threshold of 10 with potential clinical implication was proposed. However, the dynamic interaction between AAA and adalimumab and its influence on therapeutic responses remain poorly defined.

Thus, we conducted a prospective multi-center cohort study of AS patients treated with adalimumab therapy, with the following objectives: (a) identify the optimal adalimumab concentration target via exposure–response relationship models; (b) characterize the longitudinal interplay between adalimumab and AAA, as well as its clinical impact.

## 2 Methods

### 2.1 Population and study design

This real-world clinical prospective multi-center cohort study (ClinicalTrials.gov, Number: NCT04875299) was conducted at four hospitals (the First Affiliated Hospital of Soochow University, the Second Xiangya hospital of Central South University, the Third Affiliated Hospital of Soochow University, and the Shanghai Guanghua Hospital) in China between Aug 2021 and July 2023. Patients with AS diagnosed according to the modified 1984 New York criteria, who were biological-naive and starting adalimumab therapy, were enrolled. Patients who had previously received biological therapy were also eligible if a minimum of 6 months had elapsed since the last administration before initiating adalimumab. Eligible patients were aged 18 years or older. Patients with comorbidities affecting the adalimumab treatment regimen (i.e., Crohn’s disease) or influencing assessment of disease activity (i.e., gout) were excluded from the study. Patients were given 40 mg adalimumab administered subcutaneously biweekly, and any adjustments to personal treatment regimens were made based on physicians’ clinical decisions. Each patient was followed via outpatient visits in line with the next adalimumab administration. Longitudinal data, including patient characteristics, treatment regimen, and disease activity, were documented and updated at each visit. Blood samples were drawn at trough for C-reactive protein (CRP), erythrocyte sedimentation rate, adalimumab concentration, and AAA levels measurements. This study was approved by the ethics committees of the participating hospitals and all patients provided written informed consent.

### 2.2 Clinical response assessment

Disease activity was assessed at baseline and during the follow-up period using the CRP-based Ankylosing Spondylitis Disease Activity Score (ASDAS) (14). Clinical response was assessed if a patient met two criteria: (a) the presence of active disease at baseline, defined as a pre-treatment ASDAS of at least 2.1-point; and (b) completion of at least 12 weeks of therapy with adalimumab administered at 40 mg biweekly. Clinical response was defined as either a ≥2.0-point reduction in the ASDAS or a ≥1.1-point reduction that resulted in low disease activity (i.e., ASDAS < 1.3), in conjunction with normalization of CRP (i.e., < 6.0 mg/L).

### 2.3 Measurements of adalimumab and AAA

Adalimumab concentrations were measured using a validated sandwich ELISA with a couple of mouse monoclonal antibodies specific to adalimumab (GenScript, catalogue no. A01954 and A01956), as previously described (13). The adalimumab international standard (NIBSC code: 17/236) was used for calibration. The lower limit of quantification (LLOQ) was 0.0625 μg/mL. Samples with concentrations below the LLOQ were assigned a value of 0.02 μg/mL, corresponding to half of the anchor point set at 0.03125 μg/mL.

Following the analytical approach developed in our prior study (13), AAA were captured using specific functionalized beads with covalently immobilized adalimumab and measured with electrochemiluminescence using the Meso Scale Discovery platform (MSD, Meso Scale Diagnostics LLC). Rabbit-anti-adalimumab idiotype polyclonal antibodies were used as the positive AAA reference. The immunoassay exhibits high sensitivity (0.4 ng/mL), a broad calibration range (up to 10000 ng/mL), and a high drug-tolerance (10000:1 ratio of residual adalimumab versus AAA). Electrochemiluminescence signal-to-noise ratio (S/N) was used to express the magnitude of AAA. Referring to the screen cut point and confirmatory cut point predefined in our laboratory, any sample exhibiting an S/N value > 1.05 and an inhibition rate by spiked drug > 10.6% was classified as AAA positive. AAA-negative sample was assigned a value of 1.

### 2.4 Statistical analysis

Continuous variables were expressed as medians and interquartile ranges (IQR), and categorical variables were expressed as percentages. Unpaired continuous variables were analyzed using Mann-Whitney U-test or Student’s t-test as appropriate. Categorical variables were analyzed using Fisher’s exact test. Differences with a two-tailed *P* value < 0.05 was considered statistically significant. All statistics were performed with GraphPad Prism 8 (La Jolla, CA).

To depict the overall cumulative percentages of AAA occurrence, data up to the first date of observed AAA development and the last day in AAA-negative patients were included. The cumulative hazard function was examined using the Nelson-Aalen estimator in R Version 4.4.2 (R Foundation for Statistical Computing).

The following patients (n=48) were excluded from the exposure–response analysis dataset: those with pre-existing AAA, patients with a baseline disease score < 2.1, subjects who did not finish 12 week of treatment follow-up, and individuals receiving prolonged dosing interval ≥ 3 weeks. Exposure metrics included measured adalimumab concentrations and AAA-S/N values at week 2 (trough concentration after first dose administration), week 4, week 8, and the average trough concentrations between 12 and 24 weeks (at steady state). A receiver-operating characteristic (ROC) analysis was performed for adalimumab concentrations and AAA-S/N using clinical response as the classification variable. The area under the ROC curve with 95% confidence interval was calculated to evaluate the discriminative performance using GraphPad Prism 8 (La Jolla, CA, USA). Then, graphical plots were used to show the relationship between adalimumab exposure and clinical response. Patients were stratified into quartiles based on their individual exposures, with treatment response rates compared across the corresponding subgroups. The adalimumab concentration targets were identified based on the developed exposure–response model. Logistic regression models with linear link or Emax link function to describe the relationship between adalimumab exposures and the probability of achieving clinical response were developed using the glm (linear model) or gnm (Emax model) functions in R (Version 4.4.2; R Foundation for Statistical Computing). The selection of models was based on Akaike information criteria and precision of parameter estimates. The final models were evaluated by graphical plot using observed-versus-predicted response rates, alongside simulation-based diagnostics with parametric bootstrap sampling.

## 3 Results

### 3.1 Cohort and data summary

One hundred and forty AS patients were included in the study and followed prospectively. Baseline demographic and clinical characteristics are summarized in **Table 1**. All patients were treated with adalimumab monotherapy and intermittently treated with non-steroidal anti-inflammatory drugs. The median follow-up period was 169 days (IQR 99–169 days), and 711 plasma samples were available for analysis. No adalimumab was detected in samples prior to treatment, and adalimumab was undetectable in 40 samples (7.0%) after drug administration. Adalimumab concentration profiles exhibited substantial inter-individual variability (Supplemental **Figure 1A**), which may lead to inconsistent therapeutic efficacy.

**Table 1:** Baseline characteristics of the included patients.

Characteristic	Total cohort (n=140)	Responders (n=58)	Non-responders (n=34)	*P* value
Male sex, No. [%]	106 (75.7)	49 (84.5)	23 (67.6)	0.07
Age, median[IQR], y	35 (30-41)	35 (30-40)	36 (29-42)	0.97
Height, median[IQR], cm	170 (162-172)	170 (164-172)	170 (162-175)	0.87
Weight, median[IQR], kg	68 (60-75)	65 (60-75)	75 (62-80)	0.09
BMI, median[IQR], kg/m^2^	23.9 (21.7-26.0)	23.7 (21.7-26.1)	25.0 (23.1-27.6)	0.06
ASDAS baseline, median[IQR]	3.35 (2.39-4.03)	3.64 (2.86-4.22)	3.66 (3.19-4.16)	0.80
CRP baseline, median[IQR], mg/L	11.1 (4.0-25.3)	15.5 (10.3-37.5)	8.4 (5.7-22.5)	0.04
ESR baseline, median[IQR], mm/h	25 (9-52)	33 (17-58)	42 (24-64)	0.67
Albumin baseline, median[IQR], g/L	47.0 (44.4-48.1)	47.0 (44.1-48.2)	47.3 (46.1-48.0)	0.57
Previous adalimumab treatment, No. [%]	9 (6.4%)	1 (1.7%)	0 (0%)	0.99

ASDAS, Ankylosing Spondylitis Disease Activity Score; BMI, body mass index; CRP, C-reactive protein; ESR, erythrocyte sedimentation rate; IQR, interquartile range; No., number; y, years.

**Figure 1:**
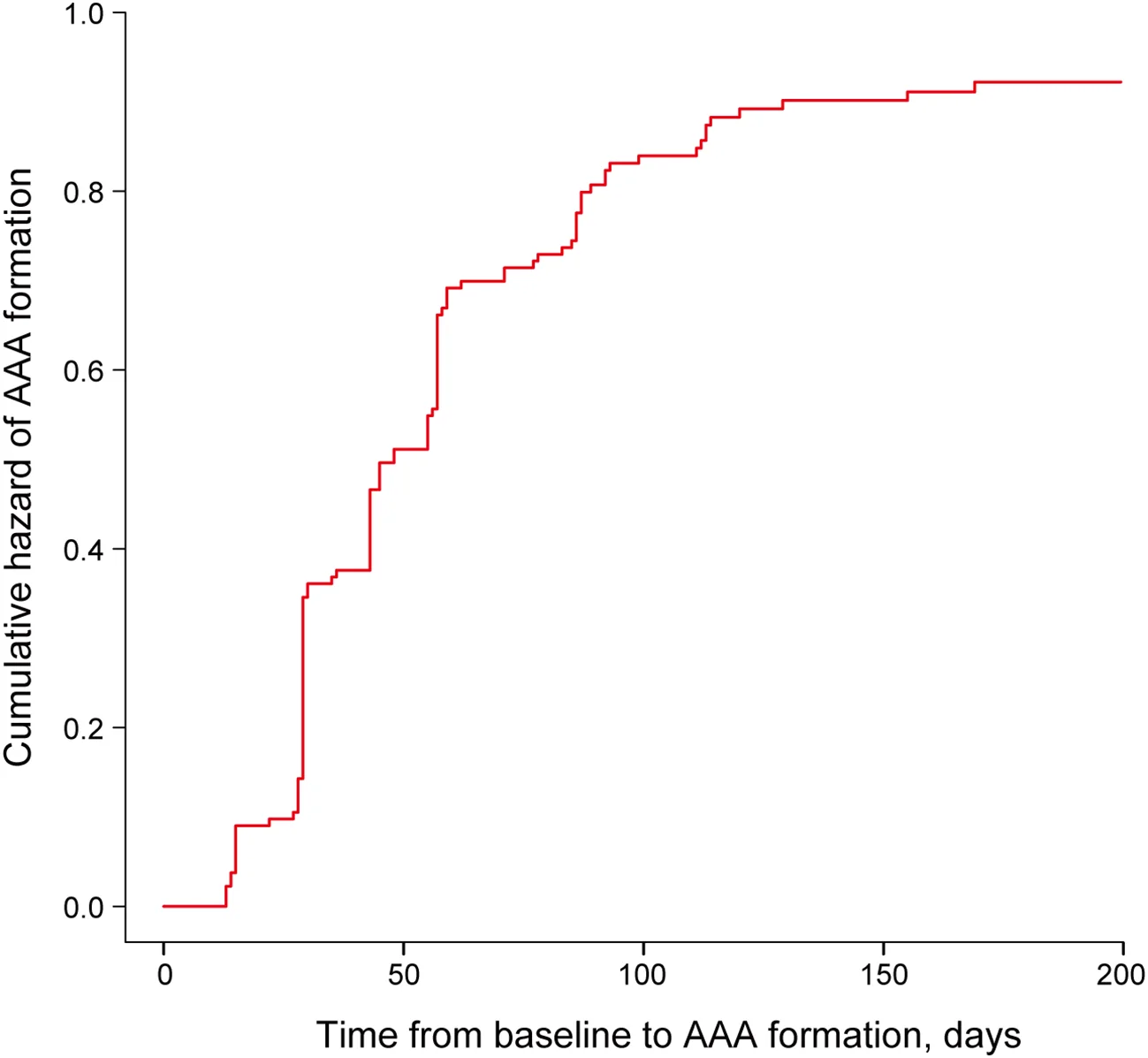
Nelson-Aalen plot showing the cumulative hazard of AAA formation. The slope of the curve indicates the hazard rate. AAA, anti-adalimumab antibodies.

Seven patients had pre-existing AAA. Among the remaining patients, 120 (90.2%) of the 133 patients had measurable AAA during the follow-up period. **Figure 1** shows the cumulative hazard profile. The median time to AAA formation was 43 days (IQR: 29–59 days). Most patients (89.2% of AAA-positive patients) developed AAA during the first three months of treatment. The inter-individual variability in AAA-S/N profiles was also substantial (Supplemental **Figure 1B**). The fitting curve shows an immune response pattern with a delayed onset followed by an escalation in intensity over repeated doses that eventually plateaus toward a maturing.

### 3.2 Positive adalimumab concentration–response relationship

A total of 48 patients were excluded from the exposure–response analysis dataset owing to pre-existing AAA (n=7), lack of active disease (n=26), incomplete follow-up (n=8), and prolonged dosing intervals (n=7). Of the remaining 92 patients, 63% (58/92) of the patients achieved clinical response. Baseline demographics are summarized in **Table 1**. Steady-state adalimumab concentrations were significantly higher in clinical responders compared with non-responders (**Figures 2A**, median: 6.2 μg/mL, IQR 4.3–7.9, vs. 1.5 μg/mL, IQR 0.4–3.8, *P* < 0.0001). Week-2 adalimumab concentrations (trough levels after the first dose) were numerically higher in responders (**Figures 2A, median**: 2.4 μg/mL, IQR 1.7–2.9, vs. 1.7 μg/mL, IQR 1.7–2.3, *P* = 0.09). Adalimumab concentrations were significantly higher in responders on week 4 and 8 (**Figure 2A**; week 4: median: 3.7 μg/mL, IQR 3.2–4.3, vs. 1.7 μg/mL, IQR 1.1–2.2, *P* < 0.0001; week 8: median: 4.3μg/mL, IQR 2.7–5.6, vs. 2.2μg/mL, IQR 1.2–3.3, *P* < 0.0001). ROC analysis demonstrated that adalimumab concentrations discriminated responders from non-responders. Of note, the area under the ROC curve for week-4 and steady-state drug level was greater than 0.85, indicating a strong discriminatory performance (**Figure 2B**).

**Figure 2:**
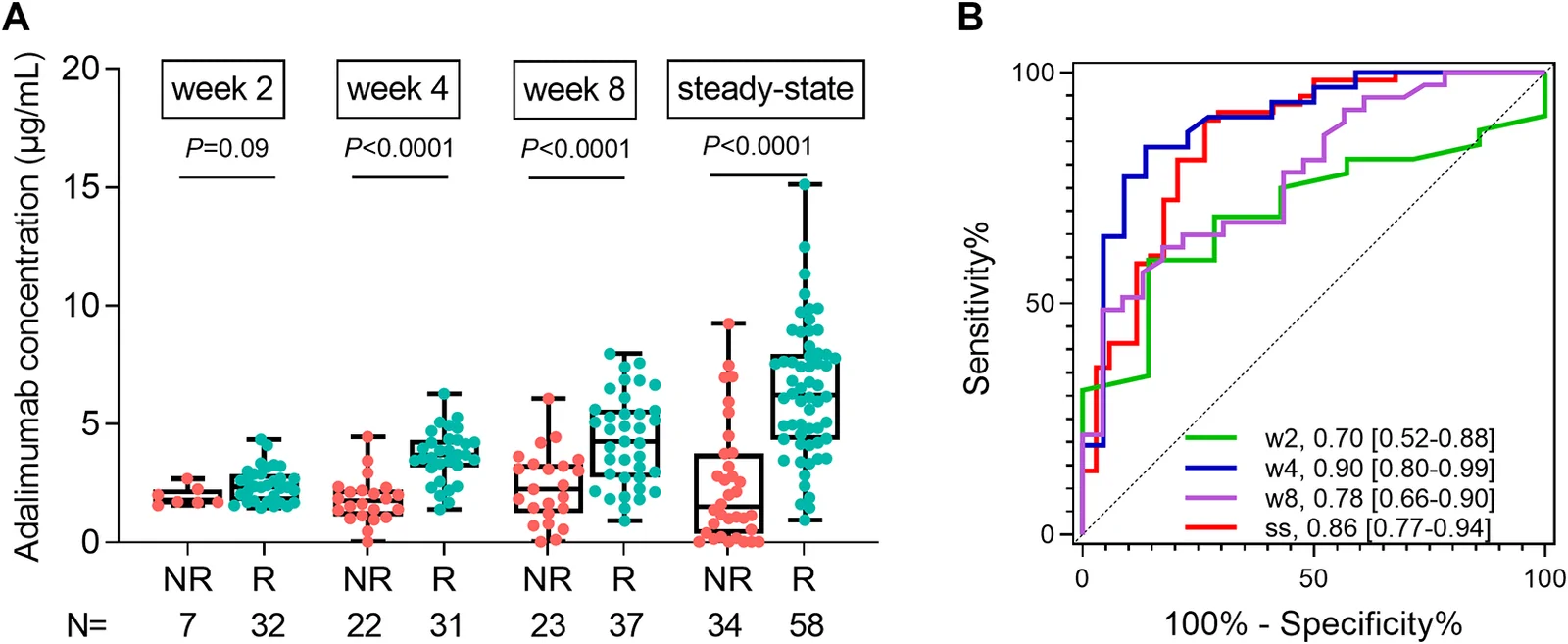
Relationships between adalimumab concentration over time and primary response. **(A)** Evolution of adalimumab concentration over time up to steady-state, separated by the primary response. Box-whisker plots showing median, interquartile range, and minimum to maximum, with all individual concentrations. **(B)** ROC curve with the AUROC and 95% confidence interval for the drug concentration at week 2 (w2), week 4 (w4), week 8 (w8), and steady-state (ss) for predicting primary response. R, responder. NR, non-responder; ROC, receiver-operating characteristic; AUROC, area under the ROC curve.

Quartile graphical analysis revealed that clinical response tended to increase with increasing adalimumab concentrations at steady-state and week 4, and the response plateaued (Supplemental **Figure 2**). A linear function was adopted to characterize the association between week-4 concentrations and clinical response, while the Emax function was utilized to describe the effect of steady-state exposure. The parameter estimates are presented in **Supplementary Table 1**. **Figure 3** depicts the progressive rise in observed- and model-predicted responder proportions as exposure levels increase. The concentration–response relationships demonstrated that a steady-state adalimumab concentration of 5.1 μg/mL corresponded to an 80% probability of achieving a clinical response (**Figure 3A**). A week-4 adalimumab concentration of 3.3 μg/mL was also associated with an 80% response likelihood (**Figure 3B**). Patients who reached the concentration target at week 4 and steady state exhibited 3.0 (relative risk, 95% CI: 1.8–5.4, *P* < 0.0001) and 1.9 times (relative risk, 95% CI: 1.4–2.8, *P* < 0.0001) more likely to achieve clinical response, respectively.

**Figure 3:**
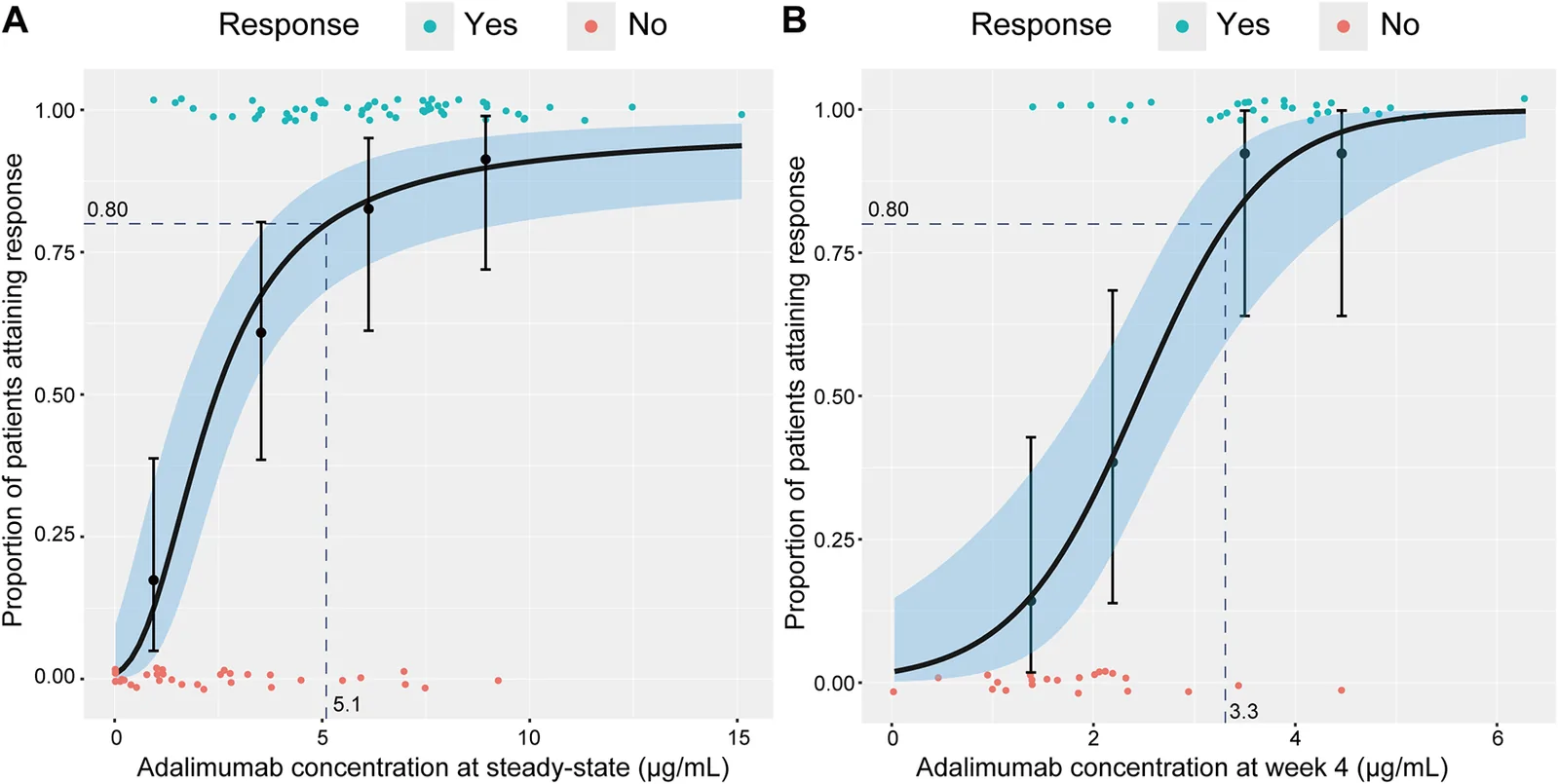
Observed and model-predicted exposure-response relationships between adalimumab concentration at steady-state **(A)** and at week 4 **(B)** and primary response. The black dots indicate the observed proportions of response, and the error bars show the 95% binomial confidence intervals. Black fitting lines indicate logistic regression model-predicted exposure-response. Shaded areas show the 95% confidence interval. Circular shapes represent the individual adalimumab concentration separated by responder (green) and non-responder (red). The dashed lines indicate the identified target concentration corresponding to the 80% probability of response.

### 3.3 Inverse association of AAA with response and drug levels

In the exposure–response analysis dataset, 84 (91.3%) patients developed AAA during follow-up. In week 2 after administration of the first dose, 6 of 39 patients (15.4%) were AAA-positive. AAA developed in 33/53 (62.3%) and 51/60 (85.0%) of patients at week 4 and week 8, respectively. Steady-state AAA-S/N was lower in responders compared with non-responders (**Figures 4A**, median: 3.4, IQR 1.8–9.7, vs. 24.9, IQR 2.3–77.4, *P* = 0.0006). Differences of AAA-S/N between responders and non-responders were also detected at week 4 and 8 (**Figure 4A**; week 4: median: 1.0, IQR 1.0–1.9, vs. 1.8, IQR 1.2–5.5, *P* = 0.003; week 8: median: 1.9, IQR 1.1–5.9, vs. 10.0, IQR 2.0–23.6, *P* = 0.004). No difference was detected at week 2. ROC analysis revealed moderate discriminatory performances for AAA-S/N at week 4, week 8, and steady-state (**Figure 4B**).

**Figure 4:**
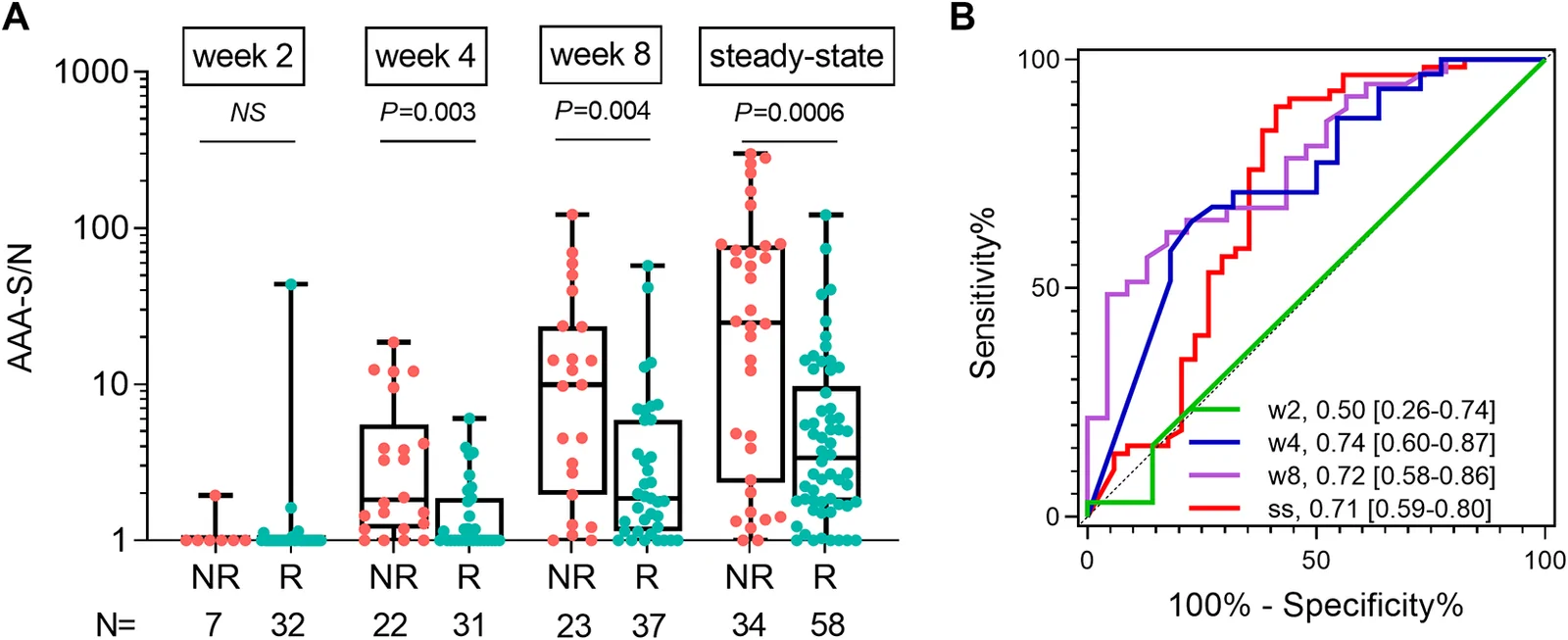
Relationships between AAA over time and primary response. **(A)** Evolution of AAA over time up to steady-state, separated by the primary response. Box-whisker plots showing median, interquartile range, and minimum to maximum, for all individual AAA-S/N. **(B)** ROC curve with AUROC and 95% confidence interval for AAA-S/N at week 2 (w2), week 4 (w4), week 8 (w8), and steady-state (ss) for predicting primary response. AAA, anti-adalimumab antibodies; R, responder; NR, non-responder; S/N, signal-to-noise ratio; ROC, receiver-operating characteristic; AUROC, area under the ROC curve.

The AAA-S/N was inversely associated with drug levels at steady-state (**Supplementary Figure 3A**). Thirty-six of 92 patients (39.1%) presented AAA-S/N values above the clinically meaningful threshold (AAA-S/N=10) established in previous study. Only two patients in the high-AAA group (S/N≥10; 2/36, 5.6%) achieved the drug target concentration (5.1 μg/mL), a proportion markedly lower than that observed in the low-AAA group (S/N<10; 40/56, 71.4%). Patients in the high-AAA group exhibited lower drug levels (**Supplementary Figure 3B**; median: 1.5 μg/mL, IQR 0.4–3.4, vs. 6.9 μg/mL, IQR 4.8–8.2, *P* < 0.0001) and poorer response rates (**Supplementary Figure 3C**, 38.8% vs. 78.6%, *P* = 0.0002) relative to those in the low-AAA group.

Based on the dynamic AAA-S/N profile, patients with week-4 AAA-positive had higher steady-state AAA-S/N (**Supplementary Figure 4A**; median: 24.5, IQR 3.2–70.9, vs. 1.4, IQR 1.0–1.8, *P* < 0.0001) and lower steady-state drug levels (**Supplementary Figure 4B**; median: 2.6 μg/mL, IQR 0.9–4.9, vs. 7.6 μg/mL, IQR 5.2–9.7, *P* < 0.0001), leading to a 1.8-fold increased risk of non-response (relative risk, 95% CI: 1.1–2.8, **Supplementary Figure 4C**).

### 3.4 Impact of pre-existing AAA on drug levels and responses

Seven patients had AAA before starting adalimumab treatment. One patient was excluded because only one dose was administered and only a single sampling time point was available. The adalimumab concentration, AAA-S/N, and disease activity of the remaining six patients are shown in **Figure 5**. A significant immune response to AAA was detected in all patients after drug administration. Elevated AAA responses were associated with the decreased or disappearance of the drug, resulting in a failure to achieve clinical response in four patients (ID127, ID94, ID8, ID73). In contrast, one patient (ID93) exhibited persistently low AAA-S/N; concurrently, the steady-state drug level reached the concentration target, ultimately leading to a clinical response. Notably, the last patient (ID76) discontinued treatment, and a reduction in AAA-S/N and an elevation in adalimumab concentration were noted.

**Figure 5:**
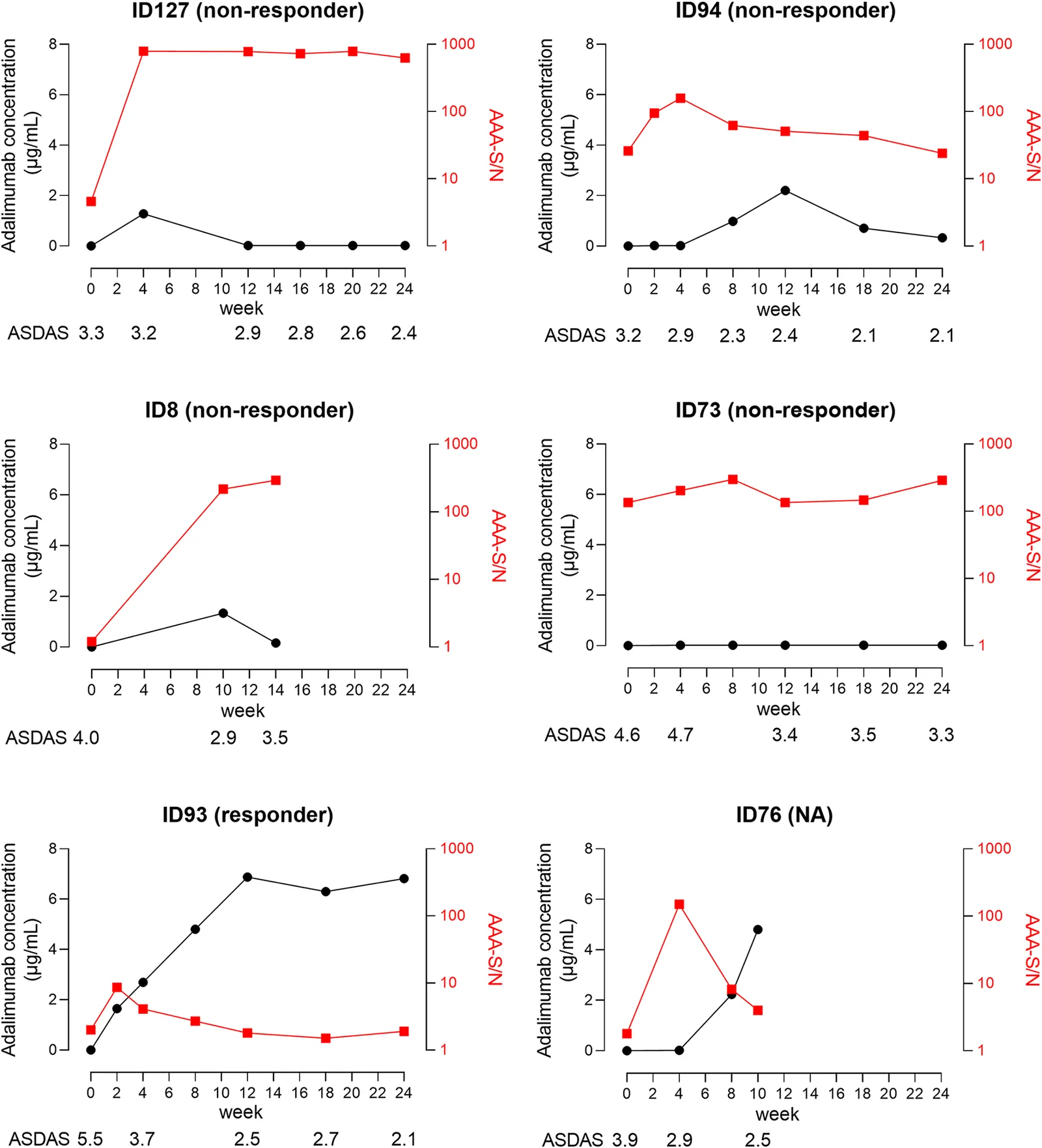
The course of changes in adalimumab concentration (black dots; left y axis) and AAA-S/N (red dots; right y axis) in single patients with pre-existing AAA. The disease activity scores were presented at the corresponding follow-up time points. ASDAS, ankylosing spondylitis disease activity score; AAA, anti-adalimumab antibodies; NA, not available; S/N, signal-to-noise ratio.

## 4 Discussion

In this real-world cohort, good exposure-response relationship models were developed to identify the optimal adalimumab concentrations at steady-state and as early as week 4 in patients with active AS. AAA levels were inversely associated with adalimumab concentrations and clinical response, indicating a potentially clinically meaningful threshold of 10 at steady-state and a risk predictor of AAA-positivity at week 4.

The identified target for the trough concentration at steady-state in AS patients (5.1 μg/mL) is consistent with other inflammatory rheumatic diseases, including rheumatoid arthritis (5–8 μg/mL) (15, 16), psoriatic arthritis (5–8 μg/mL) (17), psoriasis (3.5–7 μg/mL) (18), and Crohn’s disease (5 μg/mL) (19). Notably, the target is slightly lower than the therapeutic range previously proposed in pilot study (20). The difference may be due to differences in the measurement of adalimumab concentrations and the statistical approaches for identifying the threshold. Based on the largest prospective study to date, the optimal adalimumab concentration at week 14 was 12 μg/mL in patients with Crohn’s disease (21). In that study, the optimal threshold was determined graphically by plotting outcomes against drug concentration intervals and identifying the threshold at which increased doses were not associated with improved outcomes, defining the upper limit of the therapeutic range (22). The exposure–response model developed in this study provides a robust framework for defining an adalimumab concentration target, determined based on the clinically realistic and acceptable probability of attaining a clinical response. Naturally, the definition of clinical response also influences the threshold selection. In the present study, the definition of clinical response was slightly modified on the basis of the ASDAS criteria. Since non-normalized CRP predicts higher relapse and necessitates continuous therapy per rheumatologists’ consensus, we prioritized CRP and incorporated CRP normalization with ASDAS improvement. After dataset review, merely two patients exhibited ASDAS improvement without CRP normalization and were categorized as non-responders, which likely exerts minimal influence on the overall results. Given the favorable safety profile of adalimumab, defining an upper concentration limit based on adverse reactions is not feasible. In clinical practice, doses may be tapered for economic reasons to maintain sufficient drug exposure and sustain clinical remission.

In our study, some patients with trough concentrations below the identified target still achieved clinical response. Individual concentration targets are essential and depend on disease activity and the desired therapeutic outcomes. A higher concentration is necessary for more aggressive disease phenotypes or to achieve deeper and long-term clinical benefits (23). However, lower exposures may be appropriate for patients with low-risk disease phenotypes or patients who achieved deep remission. In clinical practice, the population-based concentration target established at 80% probability of attaining clinical response is a reference tool for evaluating whether non-responders exhibit sufficient drug concentrations (22). Admittedly, the predefined 80% probability threshold remains empirical, and alternative response probabilities would yield distinct concentration targets (24). The week-12 after initiation may be a reasonable timepoint to evaluate treatment response and determine the steady-state trough concentration (25, 26). Therefore, implementing TDM at week 12 can pinpoint the underlying factor responsible for inadequate therapeutic response and enable retention of the individualized effective concentration for responders.

Longitudinal AAA profiles in patients matched the longitudinal dynamics of humoral immunity after infection and vaccination (27, 28). Upon repeated antigen challenge, some B cells acquire a memory phenotype, which can persist for several years along with specific circulating antibodies (29). Memory B cells can be rapidly recalled and trigger a faster and stronger antibody response upon antigen re-challenge, explaining the phenomenon in patients with pre-existing AAA. Based on the longitudinal AAA profiles and the associations among drug exposure, AAA levels and clinical response, we proposed a potentially clinically meaningful AAA-S/N threshold of 10. This cutoff was previously observed in a subset of our cohort, yet the earlier exploration was solely based on pharmacokinetic analysis. This finding is supported by the highly sensitive and drug-tolerant immunoassay using the MSD-ECL platform, highlighting the importance of the choice of AAA testing for achieving reliable results for clinical decision-making (30). Comparisons of various methods are still plagued by ambiguity because a unified reference panel for AAA was not adopted (31). The development and validation of an AAA immunoassay suitable for the clinical setting, including the analytical performance and clinical performance, is a vital next step. Drug concentration can reflect the associated AAA levels due to the association between drug concentration and AAA. Drug concentrations are currently the primary testing index for TDM due to the limitations and difficulties of available methods for detecting AAA in the clinical setting. AAA testing can yield critical supplementary data to identify the drivers of insufficient drug exposure and the mechanisms underlying treatment failure. Gradually elevated ADA against infliximab determined by the same immunoassay were reported as a higher risk for loss-of-response, providing evidence to support dynamic monitoring of AAA levels (32).

We observed a high rate of AAA positivity. Of course, the reported AAA rates varied significantly (ranging between 0 and 87%) across 130 publications (33). The AAA detection assay was a significant contributor to the observed variation. The sensitivity and drug tolerance of each immunoassay determine its capacity to identify AAA positivity, thereby altering reported AAA positivity rates (34). Our study demonstrated that around 90% of patients had measurable AAA based on the MSD assay’s sensitivity of 0.4 ng/mL positive antibodies, whereas nearly 40% exhibited clinical meaningful AAA with an AAA-S/N ≥ 10, equivalent to positive antibodies concentration of 20 ng/mL (13). Thus, samples with residual drug or marginal AAA levels could yield false-negative results, regardless of whether such low-level AAA carry clinical relevance. Besides the assay, different populations also contribute to this discrepancy. For instance, concomitant use of immunosuppressors has the potential to mitigate the immunogenicity (35). Comparable high AAA positive rates have been reported in well-conducted randomized controlled studies adopting the MSD-ECLA platform (25, 36, 37).The week-4 trough concentration exhibits excellent predictive ability for primary clinical response. Early-phase adalimumab concentrations measured after the first or second dose are associated with treatment outcomes; however, this association has not been completely validated (38–42). This phenomenon may be linked to disparities in dosage schedules and the variety of concomitant medications applied in distinct populations. Notably, the time point at which significant differences emerged is closely associated with the appearance of AAA. In the POETIC study (39), week-2 adalimumab concentrations were associated with clinical remission by the end of induction, aligning with the AAA profile (i.e., AAA appeared as early as week 2 in 55% of AAA-positive patients). Thus, AAA emergence was associated with low drug concentrations and poor treatment responses. Proactive TDM of adalimumab was not recommended based on existing evidence from the PAILOT (43) and SERENE CD (44) studies. Our results provide a candidate time point to guide early proactive TDM, enabling timely regimen adjustments or addition of immunomodulators. Additional studies are warranted to determine if well-designed proactive TDM strategies can benefit patients.

Although this study did not elucidate the definitive role of TDM, our findings suggest a potential TDM strategy, as illustrated in **Figure 6**. For patients re-initiating adalimumab therapy, pre-dose AAA testing may help identify individuals at high risk of subsequent AAA development and suboptimal treatment responses. In addition to pre-dose testing, TDM at week 4 may be recommended. Low adalimumab concentration (< 3.3μg/mL) and AAA positivity are both predictive of a higher risk of primary non-response, enabling proactive intervention as early as week 4. Furthermore, TDM at week 12 may be considered in conjunction with the assessment of disease activity. In cases where the drug concentration exceeds the target (5.1 μg/mL) but no clinical response is attained, patients may be experiencing mechanism failure, warranting a therapy switch. For non-responders, adalimumab concentrations below 5.1 μg/mL indicate pharmacokinetic failure, while AAA-S/N exceeding 10 suggest immunogenicity failure. Thus, subsequent treatment regimens were modified according to the TDM results. During long-term treatment, the implementation of TDM to monitor dynamic fluctuations in drug concentration and AAA-S/N supports patient management.

**Figure 6:**
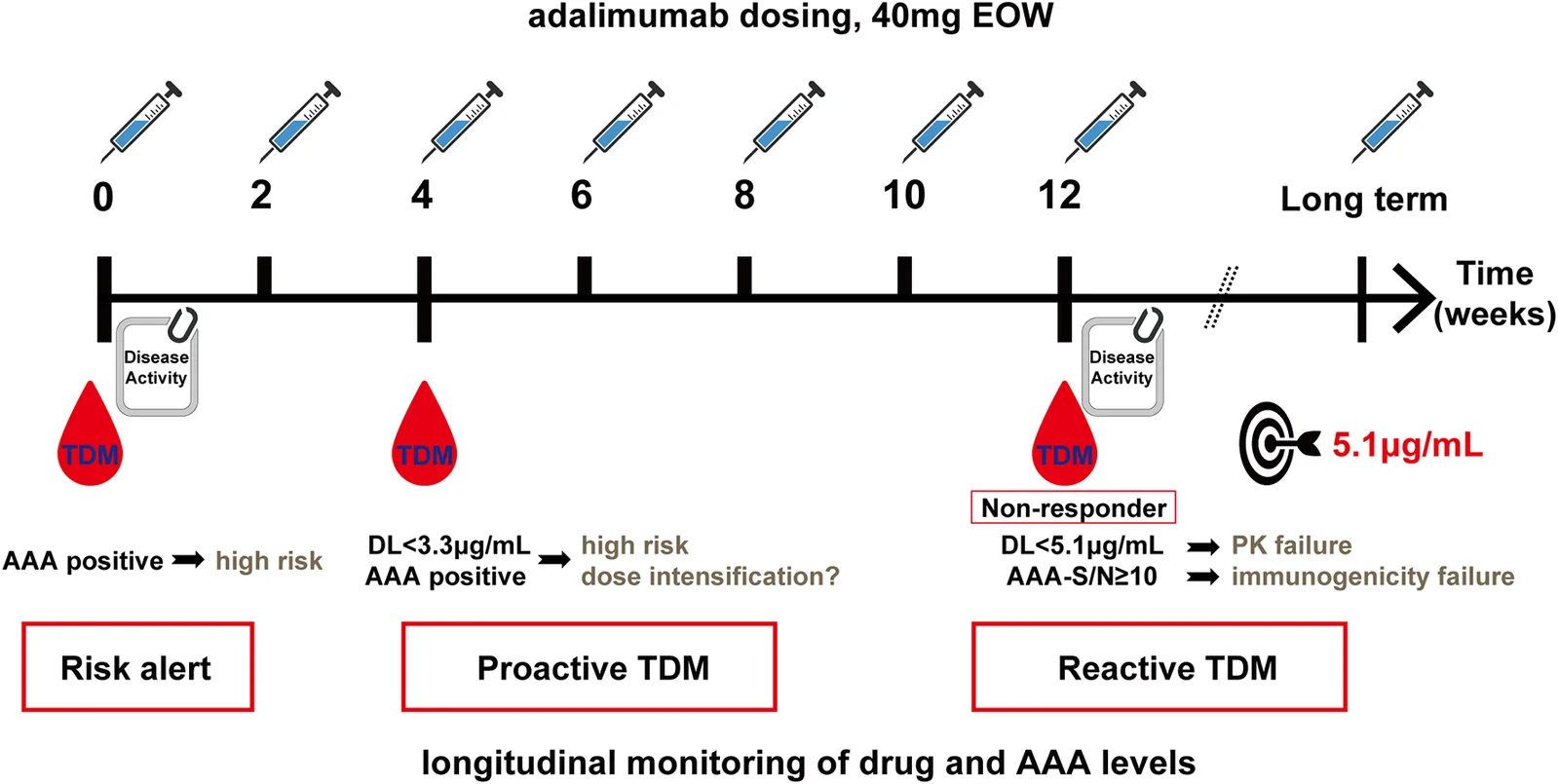
Illustrations of labeled adalimumab dosing regimen (top) and TDM–guided adalimumab treatment framework (bottom). TDM, therapeutic drug monitoring.

There are several limitations to this study. First, these findings are derived from an observational study to understand and interpret TDM results. The clinical applicability of the proposing TDM strategy should be validated by a well-designed randomized controlled trial. In addition, the AAA measurements should be unified in routine clinical settings. To address this unmet need, a chemiluminescence-based assay is being developed, considering the commercial viability, analytical performance, clinical utility, and standardized quantification protocols. Lastly, the present cohort comprised a limited number of participants, with follow-up covering the primary response. Further longitudinal studies that include patients undergoing maintenance therapy are needed to complement these findings.

## 5 Conclusions

The findings of the present study indicated that steady-state adalimumab concentrations of 5.1 μg/mL and week-4 adalimumab concentrations of 3.3 μg/mL might lead to better clinical efficacy. AAA development was correlated with diminished adalimumab exposure and reduced clinical response, highlighting the potential clinical implications of AAA measurements. Future clinical trials will be essential to validate the candidate TDM–guided adalimumab treatment framework.

## Data Availability

The original contributions presented in the study are included in the article/**Supplementary Material**. Further inquiries can be directed to the corresponding author.

## References

[1] Burmester GR Panaccione R Gordon KB McIlraith MJ Lacerda AP . Adalimumab: long-term safety in 23 458 patients from global clinical trials in rheumatoid arthritis, juvenile idiopathic arthritis, ankylosing spondylitis, psoriatic arthritis, psoriasis and Crohn's disease. Ann Rheum Dis. (2013) 72:517–24. doi: 10.1136/annrheumdis-2011-201244 22562972 PMC3595151

[2] Lagasse HAD McCormick Q Sauna ZE . Secondary failure: immune responses to approved protein therapeutics. Trends Mol Med. (2021) 27:1074–83. doi: 1016/j.molmed.2021.08.003 10.1016/j.molmed.2021.08.003 34493437

[3] Shubow S Gunsior M Rosenberg A Wang YM Altepeter T Guinn D . Therapeutic drug monitoring of biologics: current practice, challenges and opportunities - a workshop report. AAPS J. (2025) 27:62. doi: 10.1208/s12248-025-01050-9 40087239

[4] Feuerstein JD Nguyen GC Kupfer SS Falck-Ytter Y Singh S . American gastroenterological association institute guideline on therapeutic drug monitoring in inflammatory bowel disease. Gastroenterology. (2017) 153:827–34. doi: 10.1053/j.gastro.2017.07.032 28780013

[5] Kawano-Dourado L Kristianslund EK Zeraatkar D Jani M Makharia G Hazlewood G . Proactive therapeutic drug monitoring of biologic drugs in adult patients with inflammatory bowel disease, inflammatory arthritis, or psoriasis: a clinical practice guideline. BMJ. (2024) 387:e079830. doi: 10.1136/bmj-2024-079830 39467592

[6] Krieckaert CL van Tubergen A Gehin JE Hernandez-Breijo B Le Meledo G Balsa A . EULAR points to consider for therapeutic drug monitoring of biopharmaceuticals in inflammatory rheumatic and musculoskeletal diseases. Ann Rheum Dis. (2023) 82:65–73. doi: 10.1136/annrheumdis-2022-222155 35551063

[7] Zeraatkar D Pitre TS Kirsh S Jassal T Ling M Hussain M . Proactive therapeutic drug monitoring of biologic drugs in patients with inflammatory bowel disease, inflammatory arthritis, and psoriasis: systematic review and meta-analysis. BMJ Med. (2024) 3:e000998. doi: 10.1136/bmjmed-2024-000998 39574425 PMC11579540

[8] Carter PJ Quarmby V . Immunogenicity risk assessment and mitigation for engineered antibody and protein therapeutics. Nat Rev Drug Discov. (2024) 23:898–913. doi: 10.1038/s41573-024-01051-x 39424922

[9] Strand V Goncalves J Isaacs JD . Immunogenicity of biologic agents in rheumatology. Nat Rev Rheumatol. (2021) 17:81–97. doi: 10.1038/s41584-020-00540-8 33318665

[10] Papamichael K Afif W Drobne D Dubinsky MC Ferrante M Irving PM . Therapeutic drug monitoring of biologics in inflammatory bowel disease: unmet needs and future perspectives. Lancet Gastroenterol Hepatol. (2022) 7:171–85. doi: 10.1016/s2468-1253(21)00223-5 35026171 PMC10187071

[11] Gorovits B Baltrukonis DJ Bhattacharya I Birchler MA Finco D Sikkema D . Immunoassay methods used in clinical studies for the detection of anti-drug antibodies to adalimumab and infliximab. Clin Exp Immunol. (2018) 192:348–65. doi: 10.1111/cei.13112 29431871 PMC5980437

[12] Swanson SJ . What are clinically significant anti-drug antibodies and why is it important to identify them. Front Immunol. (2024) 15:1401178. doi: 10.3389/fimmu.2024.1401178 39737166 PMC11682980

[13] Ding X Xue L Wang M Zhu S Zhu K Jiang S . Dynamics and implications of anti-drug antibodies against adalimumab using ultra-sensitive and highly drug-tolerant assays. Front Immunol. (2024) 15:1429544. doi: 10.3389/fimmu.2024.1429544 39238635 PMC11374634

[14] Machado P Landewe R Lie E Kvien TK Braun J Baker D . Ankylosing Spondylitis Disease Activity Score (ASDAS): defining cut-off values for disease activity states and improvement scores. Ann Rheum Dis. (2011) 70:47–53. doi: 10.1136/ard.2010.138594 21068095

[15] Jani M Chinoy H Warren RB Griffiths CE Plant D Fu B . Clinical utility of random anti-tumor necrosis factor drug-level testing and measurement of antidrug antibodies on the long-term treatment response in rheumatoid arthritis. Arthritis Rheumatol. (2015) 67:2011–9. doi: 10.1002/art.39169 26109489 PMC4843946

[16] Pouw MF Krieckaert CL Nurmohamed MT van der Kleij D Aarden L Rispens T . Key findings towards optimising adalimumab treatment: the concentration-effect curve. Ann Rheum Dis. (2015) 74:513–8. doi: 10.1136/annrheumdis-2013-204172 24326008

[17] Vogelzang EH Kneepkens EL Nurmohamed MT van Kuijk AW Rispens T Wolbink G . Anti-adalimumab antibodies and adalimumab concentrations in psoriatic arthritis; an association with disease activity at 28 and 52 weeks of follow-up. Ann Rheum Dis. (2014) 73:2178–82. doi: 10.1136/annrheumdis-2014-205554 25115447

[18] Menting SP Coussens E Pouw MF van den Reek JM Temmerman L Boonen H . Developing a therapeutic range of adalimumab serum concentrations in management of psoriasis: A step toward personalized treatment. JAMA Dermatol. (2015) 151:616–22. doi: 10.1001/jamadermatol.2014.5479 25807311

[19] Nakase H Motoya S Matsumoto T Watanabe K Hisamatsu T Yoshimura N . Significance of measurement of serum trough level and anti-drug antibody of adalimumab as personalised pharmacokinetics in patients with Crohn's disease: a subanalysis of the DIAMOND trial. Aliment Pharmacol Ther. (2017) 46:873–82. doi: 10.1111/apt.14318 28884856 PMC5656923

[20] Ding X Zhu R Wu J Xue L Gu M Miao L . Early adalimumab and anti-adalimumab antibody levels for prediction of primary nonresponse in ankylosing spondylitis patients. Clin Transl Sci. (2020) 13:547–54. doi: 10.1111/cts.12738 31961477 PMC7214645

[21] Kennedy NA Heap GA Green HD Hamilton B Bewshea C Walker GJ . Predictors of anti-TNF treatment failure in anti-TNF-naive patients with active luminal Crohn's disease: a prospective, multicentre, cohort study. Lancet Gastroenterol Hepatol. (2019) 4:341–53. doi: 10.1016/s2468-1253(19)30012-3 30824404

[22] Wilkinson N Tsakok T Dand N Bloem K Duckworth M Baudry D . Defining the therapeutic range for adalimumab and predicting response in psoriasis: A multicenter prospective observational cohort study. J Invest Dermatol. (2019) 139:115–23. doi: 10.1016/j.jid.2018.07.028 30130616 PMC6300405

[23] Alsoud D Moes D Wang Z Soenen R Layegh Z Barclay M . Best practice for therapeutic drug monitoring of infliximab: position statement from the international association of therapeutic drug monitoring and clinical toxicology. Ther Drug Monit. (2024) 46:291–308. doi: 10.1097/ftd.0000000000001204 38648666

[24] Kantasiripitak W Wicha SG Thomas D Hoffman I Ferrante M Vermeire S . A model-based tool for guiding infliximab induction dosing to maximize long-term deep remission in children with inflammatory bowel diseases. J Crohns Colitis. (2023) 17:896–908. doi: 10.1093/ecco-jcc/jjad009 36661279

[25] Su J Li M He L Zhao D Wan W Liu Y . Comparison of the efficacy and safety of adalimumab (Humira) and the adalimumab biosimilar candidate (HS016) in chinese patients with active ankylosing spondylitis: A multicenter, randomized, double-blind, parallel, phase III clinical trial. BioDrugs. (2020) 34:381–93. doi: 10.1007/s40259-020-00408-z 32078145 PMC7211209

[26] Charles-Schoeman C Zueger P McDearmon-Blondell E Fang S Peng Y Oishi T . Real-world analysis of initial clinical response and future outcomes among patients with rheumatoid arthritis initiating and remaining on a first-line tumor necrosis factor inhibitor in the united states. Rheumatol Ther. (2026) 13:95–114. doi: 10.1007/s40744-025-00808-z 41239038 PMC12816474

[27] Almendro-Vazquez P Laguna-Goya R Ruiz-Ruigomez M Utrero-Rico A Lalueza A Maestro de la Calle G . Longitudinal dynamics of SARS-CoV-2-specific cellular and humoral immunity after natural infection or BNT162b2 vaccination. PloS Pathog. (2021) 17:e1010211. doi: 10.1371/journal.ppat.1010211 34962970 PMC8757952

[28] Turner JS O'Halloran JA Kalaidina E Kim W Schmitz AJ Zhou JQ . SARS-CoV-2 mRNA vaccines induce persistent human germinal centre responses. Nature. (2021) 596:109–13. doi: 10.1038/s41586-021-03738-2 34182569 PMC8935394

[29] Dogan I Bertocci B Vilmont V Delbos F Megret J Storck S . Multiple layers of B cell memory with different effector functions. Nat Immunol. (2009) 10:1292–9. doi: 10.1038/ni.1814 19855380

[30] Barrau M Duprat M Veyrard P Tournier Q Williet N Marc Phelip J . A systematic review on the interest of drug-tolerant assay in the monitoring of inflammatory bowel disease. J Crohns Colitis. (2023) 17:633–43. doi: 10.1093/ecco-jcc/jjac164 36301958

[31] Wadhwa M Cludts I Atkinson E Rigsby P . The first WHO reference panel for Infliximab anti-drug antibodies: a step towards harmonizing therapeutic drug monitoring. Front Immunol. (2025) 16:1550655. doi: 10.3389/fimmu.2025.1550655 40181987 PMC11965635

[32] Grasmeier MK Langmann AF Langmann P Treiber M Thaler MA Luppa PB . Dynamics of serum concentrations of antibodies to infliximab: a new approach for predicting secondary loss of response in inflammatory bowel diseases. Therap Adv Gastroenterol. (2021) 14:17562848211037849. doi: 10.1177/17562848211037849 34434255 PMC8381421

[33] Borrega R Araujo C Aguiam N Magro F Fonseca JE Danese S . Systematic review and principal components analysis of the immunogenicity of adalimumab. BioDrugs. (2021) 35:35–45. doi: 10.1007/s40259-020-00458-3 33301077

[34] van Strien J Dijk L Atiqi S Schouten R Bloem K Wolbink GJ . Drug-tolerant detection of anti-drug antibodies in an antigen-binding assay using europium chelate fluorescence. J Immunol Methods. (2023) 514:113436. doi: 10.1016/j.jim.2023.113436 36716916

[35] Ducourau E Rispens T Samain M Dernis E Le Guilchard F Andras L . Methotrexate effect on immunogenicity and long-term maintenance of adalimumab in axial spondyloarthritis: a multicentric randomised trial. RMD Open. (2020) 6:e001047. doi: 10.1136/rmdopen-2019-001047 31958280 PMC7046954

[36] Yu C Zhang F Ding Y Li Y Zhao Y Gu J . A randomized, double-blind phase III study to demonstrate the clinical similarity of biosimilar SCT630 to reference adalimumab in Chinese patients with moderate to severe plaque psoriasis. Int Immunopharmacol. (2022) 112:109248. doi: 10.2139/ssrn.4125006 36126411

[37] Feldman SR Reznichenko N Pulka G Kingo K George G Berti F . Efficacy, safety and immunogenicity of AVT02 versus originator adalimumab in subjects with moderate to severe chronic plaque psoriasis: A multicentre, double-blind, randomised, parallel group, active control, phase III study. BioDrugs. (2021) 35:735–48. doi: 10.1007/s40259-021-00502-w 34657274 PMC8520467

[38] Buffone E Gupta S Al Ibrahim B Marshall JK Halder S Tse F . Week 2 adalimumab levels predict short-term clinical remission in patients with inflammatory bowel disease. J Clin Gastroenterol. (2022) 56:794–7. doi: 10.1097/mcg.0000000000001636 34753872

[39] Ungar B Engel T Yablecovitch D Lahat A Lang A Avidan B . Prospective observational evaluation of time-dependency of adalimumab immunogenicity and drug concentrations: the POETIC study. Am J Gastroenterol. (2018) 113:890–8. doi: 10.1038/s41395-018-0073-0 29867175

[40] Mahil SK Arkir Z Richards G Lewis CM Barker JN Smith CH . Predicting treatment response in psoriasis using serum levels of adalimumab and etanercept: a single-centre, cohort study. Br J Dermatol. (2013) 169:306–13. doi: 10.1111/bjd.12341 23550925

[41] Verstockt B Moors G Bian S Van Stappen T Van Assche G Vermeire S . Influence of early adalimumab serum levels on immunogenicity and long-term outcome of anti-TNF naive Crohn's disease patients: the usefulness of rapid testing. Aliment Pharmacol Ther. (2018) 48:731–9. doi: 10.1111/apt.14943 30109889

[42] Zittan E Steinhart AH Goldstein P Milgrom R Gralnek IM Silverberg MS . Post-induction high adalimumab drug levels predict biological remission at week 24 in patients with crohn's disease. Clin Transl Gastroenterol. (2021) 12:e00401. doi: 10.14309/ctg.0000000000000401 34613952 PMC8500561

[43] Assa A Matar M Turner D Broide E Weiss B Ledder O . Proactive monitoring of adalimumab trough concentration associated with increased clinical remission in children with crohn's disease compared with reactive monitoring. Gastroenterology. (2019) 157:985–96. doi: 10.1053/j.gastro.2019.06.003 31194979

[44] D'Haens GR Sandborn WJ Loftus EV Jr. Hanauer SB Schreiber S Peyrin-Biroulet L . Higher vs standard adalimumab induction dosing regimens and two maintenance strategies: randomized SERENE CD trial results. Gastroenterology. (2022) 162:1876–90. doi: 10.1053/j.gastro.2022.01.044 35122766

