# Supplementary material for: Longitudinal monitoring of adalimumab levels and anti-adalimumab antibodies in Chinese patients with ankylosing spondylitis: a prospective cohort study
Source: Front Immunol. 2026 Oct 1;17:1893483. doi: 10.3389/fimmu.2026.1893483 (PMC13668797; doi:10.3389/fimmu.2026.1893483)
Supplement: Supplementary file 1 [file DataSheet1.docx]

***Supplemental materials***

**Title:**

Longitudinal monitoring of adalimumab levels and anti-adalimumab antibodies in Chinese patients with ankylosing spondylitis: a prospective cohort study

**Supplemental Table 1.** Parameter estimates for logistic regression models for the relationship between adalimumab exposures and clinical response

**Supplemental Figure 1.** The kinetics of adalimumab concentration (A) and AAA-S/N (B) over treatment time in individual patients, with the red line indicated the fitting curve using lowess.

**Supplemental Figure 2.** Quartile analysis depicting the exposure-response relationship between steady-state (A) or week-4 (B) adalimumab concentration and primary response.

**Supplemental Figure 3.** Inverse association of AAA-S/N with response and drug levels. (A) AAA-S/N at steady-state vs. adalimumab concentration at steady-state, separated by response. (B) Adalimumab concentrations were significantly higher in low-AAA group (AAA-S/N<10) than high-AAA group (AAA-S/N≥10). (C) Clinical response was significantly higher among those patients with low AAA, than among those with high AAA.

**Supplemental Figure 4.** Impacts of AAA status at week 4 on steady-state AAA-S/N (A), adalimumab concentration (B) and response (C).

Supplemental Table 1. Parameter estimates for logistic regression models for the relationship between adalimumab exposures and clinical response

| **Exposure metric** | **Parameter** | **Estimate** | **95% CI** |
| --- | --- | --- | --- |
| Week-4 concentration | Intercept | -3.93 | -8.03 to -2.26 |
|  | Slope | 1.61 | 0.97 to 3.27 |
| Steady-state concentration | Intercept | -4.79 | -9.80 to -2.10 |
|  | Emax | 8.40 | 6.52 to 33.54 |
|  | EC50 (μg/mL) | 1.84 | 0.45 to 44.62 |

Supplemental Figure 1. The kinetics of adalimumab concentration (A) and AAA-S/N (B) over treatment time in individual patients, with the red line indicated the fitting curve using lowess.

Supplemental Figure 2. Quartile analysis depicting the exposure-response relationship between steady-state (A) or week-4 (B) adalimumab concentration and primary response.

Supplemental Figure 3. Inverse association of AAA-S/N with response and drug levels. (A) AAA-S/N at steady-state vs. adalimumab concentration at steady-state, separated by response. (B) Adalimumab concentrations were significantly higher in low-AAA group (AAA-S/N<10) than high-AAA group (AAA-S/N≥10). (C) Clinical response was significantly higher among those patients with low AAA, than among those with high AAA.

Supplemental Figure 4. Impacts of AAA status at week 4 on steady-state AAA-S/N (A), adalimumab concentration (B) and response (C).
